# MiR-106b-5p Regulates the Reprogramming of Spermatogonial Stem Cells into iPSC (Induced Pluripotent Stem Cell)-Like Cells

**DOI:** 10.52547/ibj.3594

**Published:** 2022-07-06

**Authors:** Amir Hossein Hasani Fard, Mahmoud Valizadeh, Zohreh Mazaheri, Seyed Jalil Hosseini

**Affiliations:** 1 Men’s Health and Reproductive Health Research Center, Shahid Beheshti University of Medical Sciences, Tehran, Iran;; 2 Department of Anatomical Sciences, Faculty of Medical Sciences, Tarbiat Modares University, Tehran, Iran

**Keywords:** Computational biology, MicroRNAs, Pluripotent stem cells, Signal transduction

## Abstract

**Background:**

Recent years have brought notable progress in raising the efficiency of the reprogramming technique so that approaches have evolved from known transgenic factors to only a few miRNAs. Nevertheless, there is a poor understanding of both the key factors and biological networks underlying this reprogramming. The present study aimed to investigate the potential of miR-106b-5p in regulating SSCs to iPSC-like cells.

**Methods:**

We used SSCs because pluripotency is inducible in SSCs under defined culture conditions, and they have a few issues compared to other adult stem cells. As both signaling and post-transcriptional gene controls are critical for pluripotency regulation, we traced the expression of OSKMN. Besides, we considered miR-106b-5p targets using bioinformatic methods.

**Results:**

Our results showed that transfected SSCs with miR-106b-5p increased the expression of the OSKMN factors, which was significantly more than negative control groups. Moreover, using the functional miRNA enrichment analysis, online tools, and databases, we predicted that miR-106b-5p targeted a signaling pathway gene named *MAPK1/ERK2*, related to regulating stem cell pluripotency.

**Conclusion:**

Together, our data suggest that miR-106b-5p regulates the reprogramming of SSCs into iPSC-like cells. Furthermore, noteworthy progress in the *in vitro* development of SSCs indicates promise reservoirs and opportunities for future clinical trials.

## INTRODUCTION

Growing evidence has suggested that stem cell differentiation and reprogramming techniques are quickly expanding. The discovery of iPSCs has opened up new horizons for reprogramming technology^[1,2]^. Indeed, iPSCs and all iPSC-like cells are indispensable for the generation and banking because of their capacity conversion to any differentiated cells and tissues. iPSCs and all iPSC-like cells increase the potential for personalized cell therapies and introduce undeniable prospects for regenerative medicine, iPSC-based drug screening, disease modeling, and toxicity assessment^[3,4]^. However, somatic cells can generally be reprogrammed to iPSCs with less than 1% efficiency, and also the clinical application of iPSCs has been laden with some issues^[5]^. In this regard, SSCs have been shown to be converted into pluripotent stem cells with fewer concerns than other adult stem cells. The SSCs, postnatal germline stem cells in the testis, are capable of differentiating into sperm cells and can also be reprogrammed under characterized culture conditions^[6, 7]^. Recently, SSCs have been demonstrated to be reprogrammed into mSSCs, and pluripotency can be induced in homogeneous SSC populations without other cells. Unlike pluripotent stem cells with several issues, such as tumorigenicity and ethical concerns, these mSSCs can function as a pluripotent stem cell source free of the aforementioned problems^[8,9]^. On the other hand, various substantial functions have lately emerged for miRNAs, single-stranded noncoding small RNAs, in the regulation of pluripotency and lineage specification^[10,11]^. 

MiRNAs control protein synthesis by targeting mRNAs for translational repression or degradation at the post-transcriptional level. These molecules are phylogenetically conserved and have been validated to be influential in a wide assortment of core biological procedures, including embryogenesis and support of "stemness" among others^[12]^. Undoubtedly, the modulation of key pluripotency factors is a critical mechanism affecting reprogramming efficiency. Several miRNAs are discovered to be important regulators of stem cells, which modulate the expression of the transcription factors OSKMN, leading the somatic cells to a pluripotent state^[13,14]^. Furthermore, the specific identification of miRNA targets will help us to understand the functional role of miRNAs in pluripotent stem cells. In this field, it has been exhibited that the miR-21 expression pattern is highly correlated with *MAPK/ERK* activity during mesenchymal stem cell differentiation^[15,16]^.

According to the features of SSCs and current studies mentioned above, we hypothesized that there must be certain miRNAs as post-transcriptional regulators of SSC reprogramming. In this investigation, we selected miR-106b-5p, a member of the miR-106b-25 cluster, because recent studies have revealed that this miRNA is one of the few ones that affects the genes of signaling pathways regulating the pluripotency of stem cells^[17]^. Besides, in iPSC, the miR-106b-25 cluster is induced in early reprogramming phases, and restraint of this cluster decreases the reprogramming efficiency. *TGFBR2* and *CDKN1A* (p21) are also targets of miR-106b that have already been related to iPSC induction. Based on these findings, the present study was undertaken to evaluate the impact of miR-106b-5p on the cell reprogramming of SSCs to iPSC-like cells by 

detecting the OSKMN expression. We also studied the miR-106b-5p targets using bioinformatic methods to find out more about the possible signaling pathways involved in the SSCs reprogramming.

## MATERIALS AND METHODS

**Animal housing**

Five-day-old NMRI male mice (n = 10 each group) were used in this study. These mice had free access to food and water ad libitum and were housed under a 12-h light-dark cycle at a stable temperature and humidity-controlled room.

**Isolation and identification of SSCs**

First of all, the testis tissue fragments were minced, and then the two-step enzymatic digestion for isolating SSCs was conducted as previously described^[18]^. SSCs were characterized, and the promyelocytic leukemia zinc finger protein in the SSC-derived colonies were detected by immunocytochemistry method after seven days of culturing^[18]^.

**Preparation and transfection of hsa-mir-106b-5p plasmids**

We purchased ready-made pLV-[hsa-mir-106b] plasmid from Biosettia Inc. mir-p08. Biosettia, San Diego, CA, USA. For the amplification of plasmid, we cultured one colony of *Escherichia coli* BL21 (containing the plasmids and co-expressing *GFP* protein) in a 5-ml Luria Broth medium in a floor shaker with 1800 rpm for 16 hours. To transfect the plasmids, SSCs were seeded into six-well plate until their confluence reached 70–90%. In a microtube, 500 μl of culture medium and 7.5 µl of Lipofectamine 3000 (Invitrogen, USA) solution were mixed and incubated at room temperature for 10-15 minutes. Then 2.5 μg of the pLV-miRNA vector was added to 500 μl of the medium. After that, 250 μl of the prepared solution was added to each well. Subsequently, all wells were incubated at 37 °C for 2-4 days. Finally, the qRT-PCR technique was used to confirm transfection efficiency and *GFP* gene expression. For this purpose, the sequences of forward and reverse primers, mir-106b-5p, and U6 snRNA (as a reference gene) were downloaded from the https://www.ncbi.nlm.nih.gov/ gene website and designed using GeneRunner software (Table 1). The cDNA was synthesized according to the manufacturer’s protocols (Fermentas, USA), and the products were analyzed by electrophoresis on 1% agarose gel with a DNA ladder (1 kb).

**Table 1:** Primers used for qRT-PCR analysis

**Primer**	**Sequence (5–3)**	**Tm**
miR106b-5p-f	ACUGCAGUGCCAGCACTT	84.26
miR106b-5p-r	GGCAAAGTGCTTACAGTGC	
Stem loop	GTTGGCTCTGGTGCAGGGTCCGAGGTATTCGCACCAGAGCCAANNNNN	
U_6_	AACTGGTGTCGTGGAGTC	77.69

**Preparation and culture of mouse iPSCs**

iPSCs were purchased (Bonyakhte Stem Cells Technology, Research Center, Tehran, Iran) and generated from male NMRI mouse fibroblasts via the retroviral transfer of transcription factors *Oct4/Sox2/Klf4/c-Myc*^[19]^. The cells were used as positive control. 

**Design of study and hanging drop cell culture of the experimental groups**

This study was designed based on four experimental groups, including the SSCs, SSCs with empty vector (without any miRNA gene, Mir control), SSCs infected with miR-106b-5p (induced SSCs), and iPSCs. These experimental groups were placed in the hanging drop culture, a simple technique that suspends media by gravity and surface tension to form three-dimensional spheroids^[20]^. The hanging drops were prepared by pipetting 50 μl of media containing 70% DMEM- F12 (Gibco, UK), 10% FBS (Gibco), and 20% methylcellulose (to increase the viscosity of the solution) into a 10-cm dish at a density of 80 cells/μl. The bottom of the Petri dish was filled with approximately 3-4 ml of dH_2_O to prevent drop evaporation. After that, the lid gently was inverted over the PBS-filled bottom chamber and incubated at 37 °C/5% CO_2_/95% humidity for 48 h. The drops were monitored daily and incubated until the formation of aggregates. After a 48-h culture, aggregates were transferred to a 96-well plate pre-coated with LM agarose. In each well, 170 μl of 10% culture medium was added so that the final volume was 200 μl.

**Evaluation of miR-106b-5p and OSKMN gene expression level**

The expression level of miR-106b-5p and OSKMN genes as common pluripotent and stemness regulators were evaluated by real-time PCR. After two weeks of the hanging drop cell culture, the total RNA was extracted using the Trizol reagent from the experimental groups and then treated with DNase I (Fermentas, Germany) to eliminate genomic contamination. The cDNA synthesis was conducted by the RevertAid^TM^ First-Strand cDNA synthesis kit (Fermentas, Germany) and oligo (dT) primers. Thereupon, primers of the OSKMN genes and β-actin gene (as an internal control) were designed for PCR and RT reactions by Primer-BLAST tool (https://www.ncbi.nlm.nih.gov/tools/primer-blast/) on the NCBI database and synthesized via a commercial company (CinnaGen, Iran; Table 2). The real-time PCR techniques were performed on Applied Biosystems, StepOneTM thermal cycler (Applied Biosystems, USA) using Master Mix and SYBR Green I. The standard PCR conditions were started by a melting cycle of 5 min at 95 °C as follows: 40 cycles of melting (30 s at 95 °C), annealing (30 s at 60 °C), and extension (30 s at 72 °C). The melting curve analysis confirmed the quality of the reactions, and then the gene efficiency (logarithmic dilution series of cDNA from the samples) was specified with a standard curve. The comparative cycle threshold method (2^−ΔΔCT^) was used to examine the relative quantification of the target genes normalized against the reference gene. Then the expressions of the target genes in studied groups were examined and compared with the gene expressions in iPSCs prior to transplantation.

**Table 2:** Primers used for real-time PCR analysis

**Accession number**	**Primer**	**Sequence (5–3)**	**Product length (bp)**	**Tm**
NM_011443.4	*Sox2*	F:AGGGGAGAGAGAAAGAAAGGAG R: AATATTTGGGGGGAAGCGGAG	157	83.81
NM_028016.3	*Nanog*	F: TTATCCACTGAGCCATCTCACCA R: CCACCTTTGGTCCCAGCATTCA	188	83.04
XM_036163748.1	*Klf4*	F: CCAACACACACGACTTCCC R: CCACGACCTTCTTCCCCTCT	266	86.5
NM_001177353.1	*cMyc*	F: TAACTCGAGGAGGAGCTGGA R: GCCAAGGTTGTGAGGTTAGG	114	78.45
NM_001252452.1	*Oct4*	F: TGATTGGCGATGTGAGTGAT R: GGAGAAGTGGGTGGAGGAAG	186	81.28
NM_009608.4	*β-actin*	F: TCAGAGCAAGAGAGGCATCC R: GGTCATCTTCTCACGGTTGG	187	85

**Bioinformatic analysis, and data availability**

First, the genes of SPRPSCs were downloaded from the NCBI BioSystems database (BSID: 1026136; https://www.ncbi.nlm.nih.gov/). Next, the functional miRNA enrichment analysis of the genes was performed using the FunRich software (version 3.1.3) available for public access. Thereafter, we determined the potential genes, which hsa-miR-106b-3p and hsa-miR-106b-5p would target by means of online tools and databases (http://www.targetscan.org/ and http://mirwalk.umm.uni-heidelberg.de/). Therefore, we generated a scalable Venn diagram to find common genes targeted by both arms of miR-106b. The common target genes and the genes of SPRPSCs ultimately were represented in a Matrix table (pair-wise comparison). Besides, we showed a Heatmap image of the common targets based on the human proteome map of the FunRich.

**Statistical analysis**

Statistical analysis of differences was carried out using one-way analysis of variance (ANOVA) and Tukey’s post-test on GraphPad Prism version 8.0.0 for Windows (Graph Pad Software, San Diego, CA). Six biological replicates were used in each experimental group. The *p* values <0.05 were considered statistically significant for each experiment, and error bars represent ± SD.

## RESULTS

**Characterization of SSCs**

**Fig. 1:**
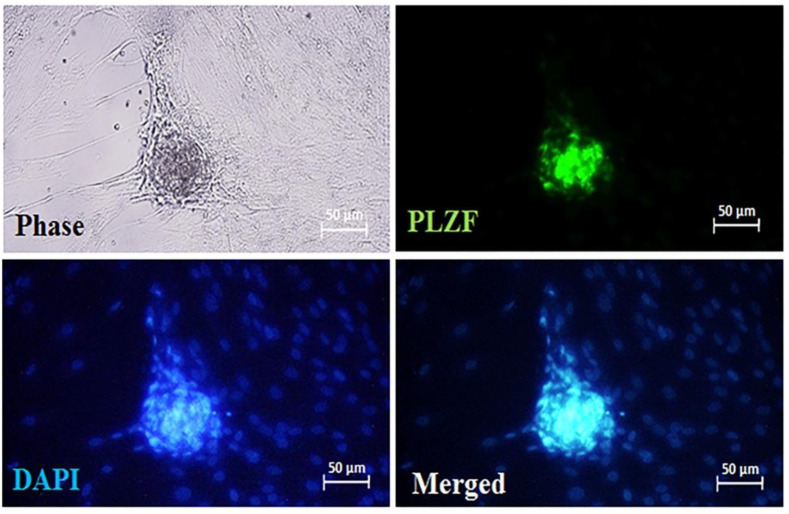
SSCs characterization. After seven days of culturing, the SSCs colonies were derived from the cultured cells. Immunocytochemistry results revealed the expression of promyelocytic leukemia zinc finger protein as an undifferentiated spermatogonia marker by SSCs, and DAPI (chromatin marker) indicated the location of cell nuclei.

PLZF, also known as ZBTB16 (zinc finger and BTB domain-containing 16) is a consensus marker for undifferentiated spermatogonia^[21]^. Immuno-cytochemistry analysis illustrated the expression of this factor in the colonies derived from the cultured cell suspensions (Fig. 1). 

**Confirmation of transfected cells**

**Fig. 2:**
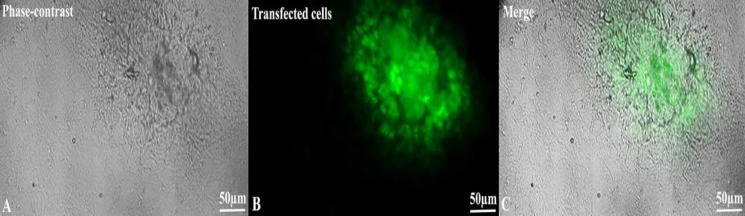
Evaluation of transfection. *GFP* protein expression of the transfected SSCs was followed under a phase-contrast microscope after 48 hours. The result of 61.53% efficiency confirmed the transfection. Images of phase-contrast (A), *GFP*-positive (*GFP+*) cells (B), and merged (C).

Colonies (n = 16) of SSC per six-well plate were transfected for 48 h (the total mean of the colony was 26). To corroborate the transfection of SSCs with Mir control and the expression vector containing miR106b-5p, the cells were observed under a phase contrast microscope. The expression of *GFP* protein as a reporter gene was detectable after two weeks. The efficiency of transfection was 61.53% per repetition (Fig. 2).

**Gene expression signatures of pluripotency and miR-106b-5p**

**Fig. 3:**
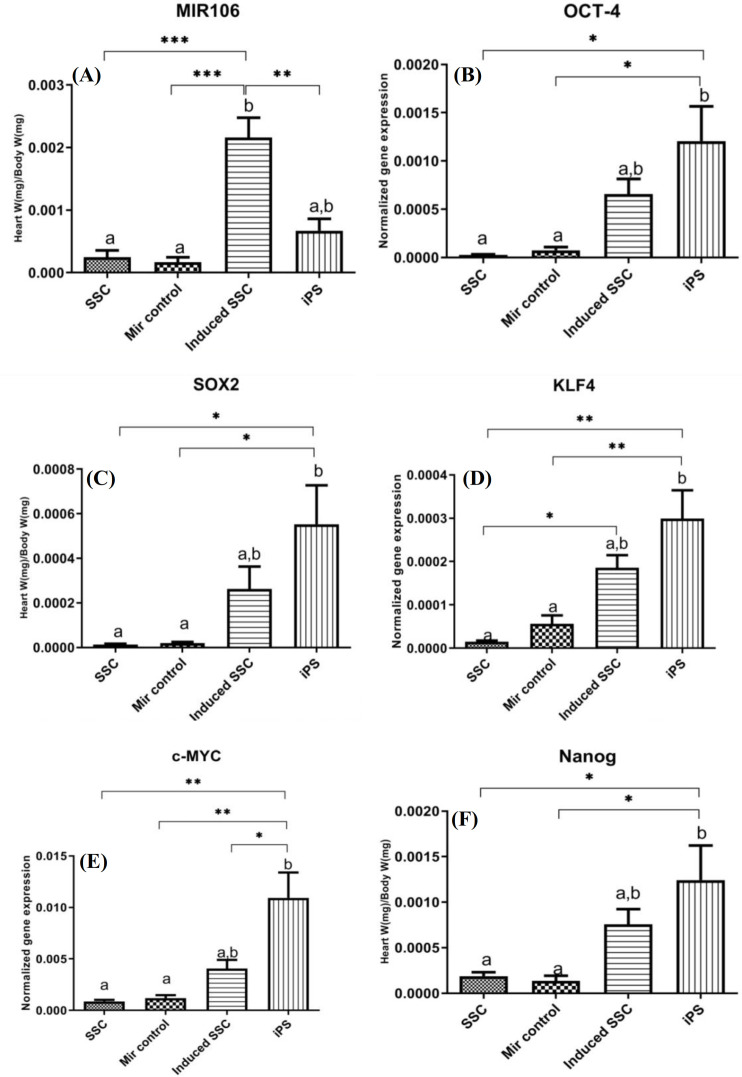
Molecular assessment. Analysis of miR-106b-5p and OSKMN expression in the experimental groups. (A) The results first confirmed the expression of the miR-106b in the induced SSCs, which attested robust transfection (*p* = 0.0003). In addition, *Oct-4* (B; *p* = 0.0088), *Sox-2* (C; *p* = 0.0162), *Klf-4* (D; *p* = 0.0024), *c-Myc* (E; p = 0.0021), and *Nanog* (F; *p *= 0.0176) as pluripotency markers were significantly expressed in the induced SSC groups. Significant differences for Tukey’s multiple comparisons test: ^***^p ≤ 0.001; ^**^p ≤ 0.01; ^*^p ≤ 0.05. The error bars represent ±SD.

Real-time PCR was utilized to determine the expression of miR-106b-5p and OSKMN, a subset of pluripotency markers, in all study groups. The findings first strengthened that the SSCs were transfected with miR-106b-5p (fold change = 15.96). Furthermore, the mean expression of the *Oct-4* (fold change = 10.17), *Sox-2* (fold change = 11.90), *Klf-4* (fold change = 4.76), *c-Myc* (fold change = 3.43), and *Nanog* (fold change = 6.73) genes in iPSC and induced SSC groups were significantly more than the negative groups. Interestingly, the induced SSC experimental groups showed similar properties to the iPS groups regarding the expression of OSKMN genes (Fig. 3).

**MAPK1/ERK2 a target for pluripotency regulation**

**Fig. 4:**
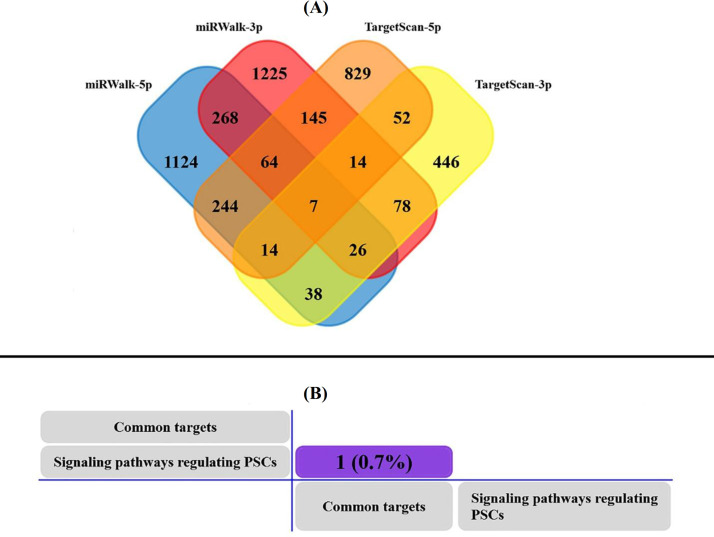
(A) Venn diagram of the functional miRNA enrichment analysis showing overlaps between miRWalk and TargetScan genes as possible targets of miR-106b-5p, which consists of seven common genes (B). To find the common genes between these seven genes and SPRPSCs, we used Matrix table (pair-wise comparison). MiR-106b targeted the *MAPK1/ERK2* gene.

**Fig. 5:**
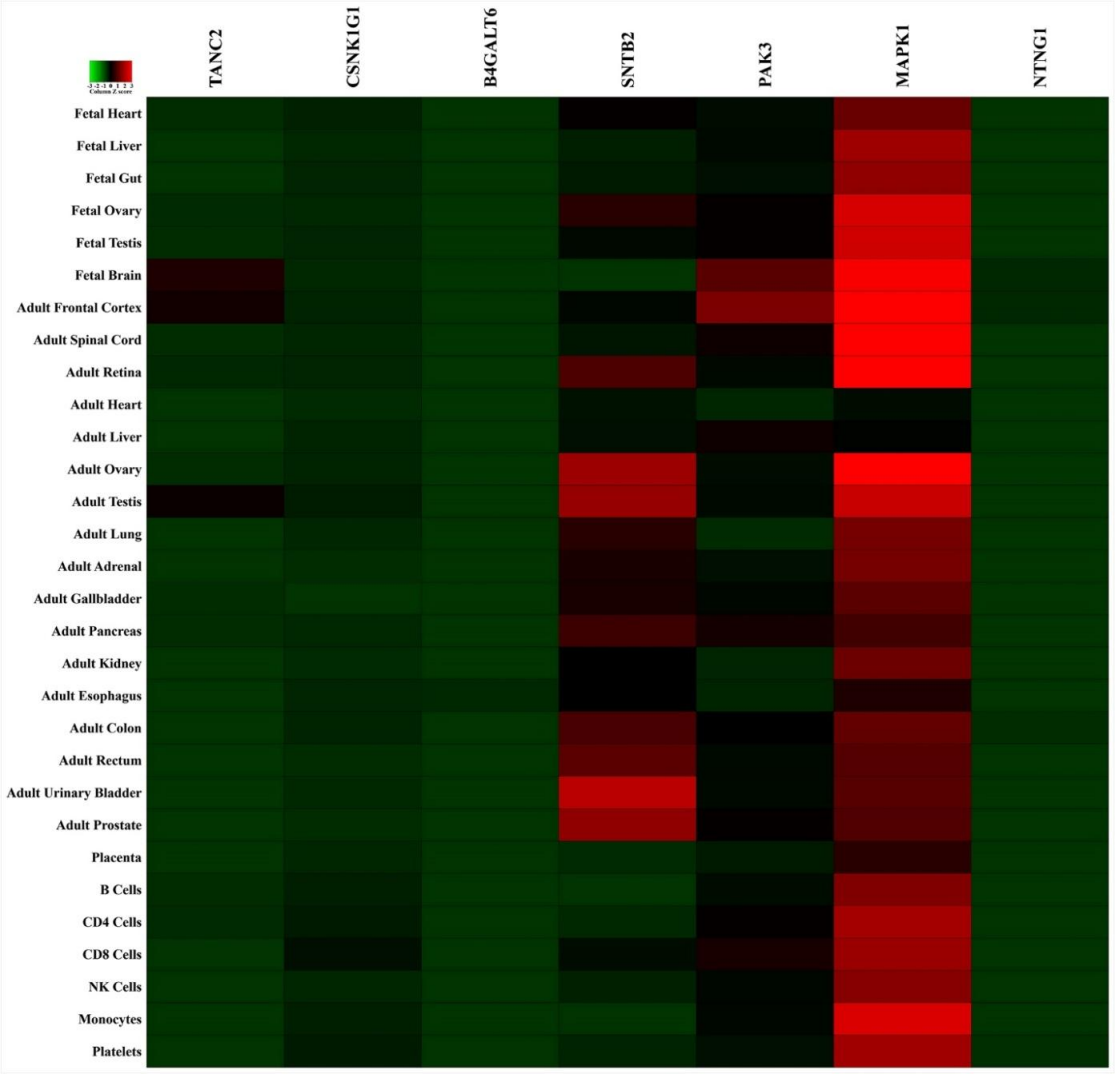
Heat map representing different expressions of *MAPK1 *(*ERK2)* in the human proteome map, especially in the adult and fetal testis (green—lowest abundance and red—highest abundance).

The functional miRNA enrichment analysis revealed the miRNAs targeting the genes of SPRPSCs (Target score: ≥50%). Among the miRNAs found via FunRich, miR-106b was selected based on its potential. As the scalable Venn diagram is illustrated, miR-106b targeted seven common genes (Fig. 4A). Consequently, comparing these common genes with those of SPRPSCs showed that miR-106b targets *MAPK1*/ *ERK2* gene (Fig. 4B and Table 3). In addition, the Heatmap image exhibited different expressions of *MAPK1/ERK2* in the human proteome map, especially in the fetal and adult testis (Fig. 5).

## DISCUSSION

Over the past years, much effort has been directed towards screening small molecules to improve reprogramming efficiency and create new methods for iPSC derivation. MiRNAs have played a key role in regulating pluripotency and lineage specification by modulating gene expression at the post-transcriptional level, resulting in iPSCs generation from various cell types^[22-26]^. Previously, He and colleagues^[27]^ explored the expression, role, and targets of miRNA-20, miRNA-106a, and miRNA-93 in SSCs. Their results provided that these endogenous small RNA molecules regulate SSCs and have vital implications on offering new therapeutic targets. In another study, several authors have shown that miRNA-20 and miRNA-106a promote the renewal of mouse SSCs at and *CCND1*, thereby regulating SSC fate determinations^[28]^. As a preliminary proof-of-concept, the present investigation indicates that miR-106b-5p is a practicable factor in reprogramming SSCs into iPSC-like cells. Herein, miR106b5p-infected SSCs expressed a subset of pluripotency markers (OSKMN), almost the same as iPSCs. In addition, the bioinformatics analysis in this study predicted that miR-106b targeted *ERK2*, a gene of the signaling pathways regulating stem cell pluripotency. Overall, these data suggest that miR-106b-5p regulates the reprogramming of SSCs into iPSC-like cells by targeting the *ERK2* gene.

Transcriptional networks involving a set of pluripotent transcription factors control and sustain the pluripotency of stem cells. These pluripotent genes stimulate or suppress downstream gene expression, inducing the event of some signaling pathways and regulate the pluripotency of stem cells. It has previously been demonstrated that signaling and post-transcriptional gene control are both essential for pluripotency regulation, but it remains poorly known how they are incorporated to affect cell identity. In pluripotent cells, phosphorylation as a pervasive form of cell signaling plays a crucial role in controlling cell identity by relaying signaling of the growth factor via chief pathways. The *ERK2* is one of the best-characterized MAP kinase pathways that phosphorylates *Klf4*, *OCT4*, *SOX2*, and *NANOG*^[29-33]^. For instance, the nuclear export of *KLF4* requires *ERK* activation, and the phosphorylation of *KLF4* by *ERK* commences the interaction of *KLF4* with the nuclear export factor *XPO1*, resulting in the export of *KLF4*. The mutation of the ERK phosphorylation site in *KLF4* prevents *KLF4* nuclear export, decreases Nanog and *KLF4* mRNA levels and prevents differentiation^[34]^. In light of these findings and our results, it can be inferred that *ERK2* activation induced by miR-106b-5p initiates the reprogramming of SSCs to a pluripotent state. 

As Yamanaka^[35]^ first generated iPSCs in 2006, its clinical applications have been laden with some issues such as tumorigenicity. The different expression level of *c-Myc*, frequently reported as a tumorigenesis factor, is one of the iPSCs therapeutic application hurdles. Despite inducing miR-106b∼25 cluster, *c-Myc* is activated as a proto-oncogene, giving rise to several cancers^[36-39]^. Herein, we highlight that the induced SSCs express less *c-Myc* than iPSCs; therefore, these iPSC-like cells have the potential to be a considerable alternative as a pluripotent stem cell source. Furthermore, we have previously performed an *in vivo* study on the tumorigenicity of SSCs infected with miR-106b-5p^[40]^.

**Table 3:** List of common miR-106b targeted genes

**Identifier**	**Official full name**	**Mean RPKM in testis**
*MAPK1*	mitogen-activated protein kinase 1	23.469 ± 3.817
		
*CSNK1G1*	casein kinase 1 gamma 1	6.568 ± 1.596
		
*TANC2*	tetratricopeptide repeat, ankyrin repeat and coiled-coil containing 2	5.949 ± 0.57
		
*SNTB2*	syntrophin beta 2	5.22 ± 0.634
		
*B4GALT6*	beta-1,4-galactosyltransferase 6	2.103 ± 0.696
		
*PAK3*	p21 (RAC1) activated kinase 3	1.279 ± 0.334
		
*NTNG1*	netrin G1	0.129 ± 0.029

Regarding our results, we observed no palpable tumors two months after injection in the dorsal subcutaneous space. However, histological examination of the tissue sections showed tumorigenicity of iPSC group injection^[40]^. Based on the empirical evidence assembled in the paper, we tend to conclude that the SSCs can be considered as a noticeable candidate for cellular reprogramming strategies. Moreover, miR-106b is a key molecule in the SSCs reprogramming that upregulates pluripotency-associated factors. Collectively, our results showed that miR-106b-5p regulates the reprogramming of SSCs into iPSC-like cells by targeting the *ERK2* gene. 

## DECLARATIONS

### Acknowledgments

This study was financially supported by Men’s Health and Reproductive Health Research Center, Shahid Beheshti University of Medical Sciences, Tehran, Iran. We acknowledge the Histogenotech Company and its staff who provided insight and expertise that greatly assisted the research.

### Ethical statement

All experiments were approved by the Ethics Committee of Shahid Beheshti University of Medical Sciences and followed the Declaration of Helsinki (Approval ID: IR.SBMU.REC1398.074).

### Data availability

The datasets used and/or analyzed during the current study are available from the corresponding author on reasonable request.

### Author contributions

AHHF: proposed the work, performed experimental works and data collection and contributed to manuscript writing and editing; MV: performed bioinformatics work and contributed to manuscript writing and editing; ZM: proposed the idea; SJH: proposed the work. All authors have read and approved the final version of manuscript.

### Conflict of interest

None declared.

### Funding/support

This study was supported by a research grant from Men’s Health & Reproductive Health Research Center (MHRHRC), Shahid Beheshti University of Medical Sciences, Tehran, Iran.
